# Temperature during Pregnancy, Labor and Childbed

**Published:** 1871

**Authors:** S. Brandeis

**Affiliations:** Louisville, Ky.


Art. III.—*Temperature During Pregnancy, Labor and Childbed.* By S. Brandeis, M.D., Louisville, Ky.

Mr. Squire, in London, published an article under the above title, in the *London Lancet,* in 1867.

During pregnancy, the temperature is generally somewhat increased, after the sixth month, to an average of 99 deg.

During a normal parturition, the temperature was generally but little higher, the maximum being 99.9 deg., and the minimum 98.9 deg.

In the first twenty-four hours after labor, there is a fall of temperature observed. With the secretion of milk there is a rise, which gradually disappears as soon as the same is established. The highest figure was 103.3 deg., on the tenth day. Sleep seemed to diminish the temperature.

For the period of involution, solid food and warm diluent drinks will favor the rising of temperature ; the contrary is done by alcoholic drinks. With reference to the use of the latter, Squire gives the following indications in connection with thermometrical conditions:

1st.—As long as the temperature is rising and the lacteal secretion not fully established, alcoholic stimulants may be useful, and even necessary.

2d.—When the secretion of milk is copious and the temperature high, stimulants are unnecessary, and seem to be even injurious.

3d.—When the secretion of milk is profuse and the temperature low, alcoholic drinks are required.

Dr. Crede, Professor of Midwifery, in Leipsig, read a paper on Thermometrical Investigations in the Puerperal State, at the Convention of German Naturalists and Physicians, at Dresden, in 1869.

Since the beginning of 1866, every lying-in woman in his clinic was the subject of thermometrical investigation at least twice a day. He prepared numbers of tables and curves, and finally arranged his cases in the following groups:

1st.—Temperature never above 38 Celsius (bloodheat); perfectly normal cases.

2d.—Sudden rise on the third day, with an early fall to the normal temperature. Afflux of milk to the mammæ, sore nipples, slight contusions, and injuries of the genitals; painful after-pains.

3d.—Rise of temperature on the fourth, fifth, and sixth days, with an early fall to the normal temperature. Constipation most frequently; no material changes; perhaps some indiscretion in diet or physical excitement.

4th.—Rise on the third and fourth days; persistently high for fourteen days; then slow return to normal temperature; complications of slight puerperal affections, with disturbances of digestion; slight exudation surrounding the uterus.

5th.—Rise of temperature on the second, third and fourth days, persisting in its height for three or four days; then a rapid fall. Diphtheritic deposits upon the mucous lining of the genitals; slight endometritis.

6th.—Rise from the sixth to eighth day; duration from two to eight days; then a sudden fall; softening and detachment of thrombus in the uterine cavity; general condition scarcely disturbed.

7th.—Rapid and considerable rise of temperature right after labor, as on the first and second days, with long duration and without essential fall. Malignant diseases, terminating either fatally or with slow and exhausting convalescence; endometritis, peritonitis, perimetritis, phlebitis, etc., always with various and serious complications, with extensixe and destructive collections of pus and sanies.

In this manner the author succeeded in demonstrating the intimate connection between a rise in the temperature and disease in the puerperal state.—*Richmond and Louisville Med. Jour.*

